# Pectin Characteristics Affect Root Growth in Spinach under Salinity

**DOI:** 10.3390/plants11223130

**Published:** 2022-11-16

**Authors:** Jia Liu, Victoria Otie, Asana Matsuura, Kashiwagi Junichi, Muhammad Irshad, Yuanrun Zheng, Haruyuki Fujimaki, Ping An

**Affiliations:** 1 Arid Land Research Center, Tottori University, 1390 Hamasaka, Tottori 680-0001, Japan; 2 Department of Soil Science, Faculty of Agriculture, Forestry and Wildlife Resources Management, University of Calabar, P.M.B. 1115, Calabar 540271, Nigeria; 3 Faculty of Agriculture, Shinshu University, 8304, Minamiminowa-Village, Kamiina-County Nagano, Nagano 399-4598, Japan; 4 Graduate School of Global Food Resources, Hokkaido University, Kita 9, Nishi 9, Kita-ku, Sapporo 060-0809, Japan; 5 Department of Environmental Sciences, Abbottabad Campus, COMSATS University Islamabad (CUI), Abbottabad 22060, Pakistan; 6 Key Laboratory of Resource Plants, West China Subalpine Botanical Garden, Institute of Botany, Chinese Academy of Sciences, Beijing 100093, China

**Keywords:** plant cell wall, pectin, HG:RG-I ratio, viscosity, *Spinacia oleracea*, salinity stress

## Abstract

In understanding the role of root cell wall mechanisms in plant tolerance to salinity, it is important to elucidate the changes in the pectin composition and physical properties of the cell wall. Two salt-sensitive (Helan 3 and Prius β) and one salt-tolerant (R7) spinach cultivars were used to investigate the pectin polysaccharides, the characteristics of pectin, including the degree of pectin methy-lesterification, the HG:RG-I ratio, neutral side chains (galactan/arabinangalactan), and elasticity and viscosity parameters in the root elongation zone under salinity. Root growth was inhibited by salinity, whereas the root diameter was thickened in all cultivars. Salinity significantly reduced cell wall extensibility in all cultivars, and increased cell wall viscosity in Helan 3 and R7 relative to Prius β. Pectin was significantly increased under salinity stress. Cell wall viscosity was affected by pectin due to the molar proportion of uronic acid and/or pectin characteristics (HG:RG-I ratio). The molar proportion of uronic acid in pectin was reduced in Helan 3 and R7 compared with Prius β. The length and degree of pectin methy-lesterification of neutral side chains were significantly decreased in the R7 cultivar, with no significant changes in the other two cultivars. Demethylation of pectin could alter root growth and boost salt tolerance in the R7 cultivar. In this study, it is shown that cell wall pectin played important roles in regulating the root growth of *Spinacia oleracea* L. under salinity stress.

## 1. Introduction

Soil salinity is an important environmental problem for more than 800 million hectares of land, which results in osmotic stress, ionic imbalances, ion toxicity, oxidative damage and complex effects on the physiology and metabolism of plants, including spinach (*Spinacia oleracea* L.) [1,2]. The growth of most spinach crops is reduced when the soil salinity exceeds 4 dS/m of electrical conductivity, which is equivalent to 40 mM sodium chloride [3,4]. Although excessive salts are toxic to salt-sensitive plants, some cultivars in spinach may be salt tolerant once adapted to a moderate saline stress. Exposure to high salt concentrations adversely affects crop performance due to salinity-induced nutritional imbalance. Studying the physiological responses of cultivars of spinach with different levels of salt tolerance is a useful tool for understanding the mechanisms underlying plant responses to salinity.

The root cell wall is a major storage site for several environmental pollution problems, including salinity. It acts as a protective barrier of protoplasts by trapping toxic substances to reduce cellular damage mainly caused by salts or other trace metals [5]. Its important role in plant resistance to salinity stress could be attributed to the interaction with salts present in plants and the soil [6,7,8]. The cell wall matrix is composed of pectin, hemicellulose, and cellulose. Pectin is composed of homogalacturonan (HG) and rhamnogalacturonan I (RG-I) [9]. The main changes in the cell wall following salinity stress were found in the pectin sugar composition, pectin characteristics such as HG:RG-I and the degree of methyl esterification [10,11,12]. The synthesis of galactose and arabinose side chains are also considered to contribute to maintaining cell wall integrity under salinity stress [10,13].

The plant cell wall is essential for the strength, growth and development of plants [14]. The cell walls of spinach contain phenolic acids (ferulic, p-coumarics, and diferulic), which are bound to polysaccharide compounds, including pectin. These affect the physical properties of cell walls by increasing the calcium cross-links between homogalacturonans, resulting in the stiffening of the pectin gel and primary cell walls [15]. Xiong et al. [16] reported that cell expansion in rice seedlings cultured in the absence of Ca^2+^ was still regulated by pectin. Huang et al. [11] reported that a modified pectin structure can provide a different strength for the cell wall architecture. However, there are few studies on the structural changes of cell wall pectin under salinity stress. Our previous research revealed that cell wall pectin played important roles in cell wall extension in both *Spinacia oleracea* and *Suaeda salsa* under salinity, and that the salt tolerance of *S. oleracea* was affected by pectin [17].

Spinach (*Spinacia oleracea* L.), being a well-known leafy vegetable with various salinity tolerance levels in different cultivars, has been recently reported [2,17,18,19,20]. In this study, we investigated the salinity tolerance of three spinach cultivars with a focus on pectin content, such as: pectin polysaccharides, the degree of pectin methy-lesterification (PMD) and pectin-related wall parameters in the cell walls.

## 2. Results

### 2.1. Root Growth

**Figure 1:**
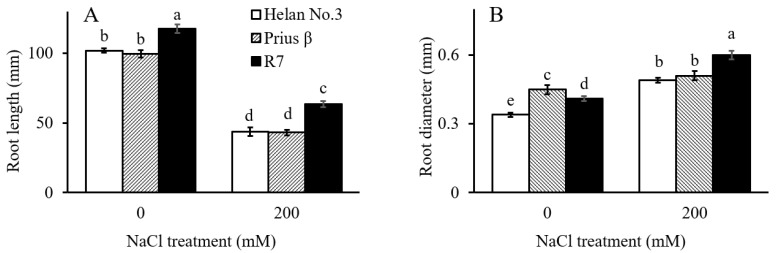
Final root length, relative root length (**A**) and root diameter (**B**) of Helan 3, Prius β and R7 in 0 and 200 NaCl treatments. Data are mean ± S.E. (*n* = 6). Different letters indicate significant differences (*p* < 0.05).

Salinity significantly inhibited root elongation in all spinach cultivars (Figure 1A). Root growth across the cultivars was significantly inhibited under 200 mM NaCl treatment. This inhibition was more pronounced in Helan 3 and Prius β, compared with R7, which was a more tolerant cultivar. The diameter of the roots of all three cultivars increased significantly under salinity (Figure 1B). There was a 44% and 46% increase in rooting diameter in Helan 3 and R7 under salinity stress, respectively, whereas a 13% increase was observed in Prius β (Figure 1B).

### 2.2. Root Cell Wall Extensibility and Viscosity

**Figure 2:**
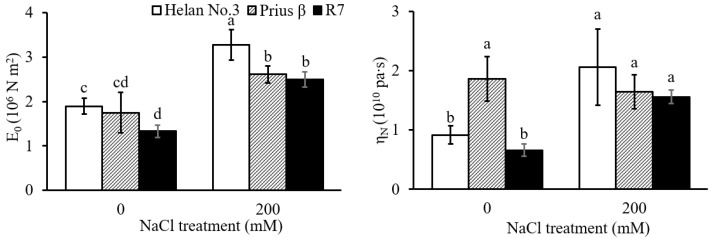
Elastic moduli (E_0_) and viscosity coefficient (η_N_) of the root cell wall in the elongation zone in Helan 3, Prius β and R7 in 0 and 200 mM NaCl treatments. Data are mean ± S.E. (*n* = 15–24). Different letters indicate significant differences (*p* < 0.05).

The elastic moduli of E_0_ in root elongation zone in all three cultivars increased significantly under salinity stress (Figure 2). The E_0_ in the salt-sensitive cultivar Helan 3 was significantly higher compared with the salt-tolerant cultivar R7, whether under 0 or 200 mM NaCl. The viscosity coefficient, η_N_, was significantly increased in the sensitive Helan 3 cultivar and tolerant R7cultivar. However, there was no significant change in Prius β (Figure 2). Meanwhile, in 0 mM NaCl treatment, the viscosity coefficient of Prius β was significantly higher than that of the other two cultivars (Figure 2). 

### 2.3. Chemical Composition of Root Cell Wall

**Figure 3:**
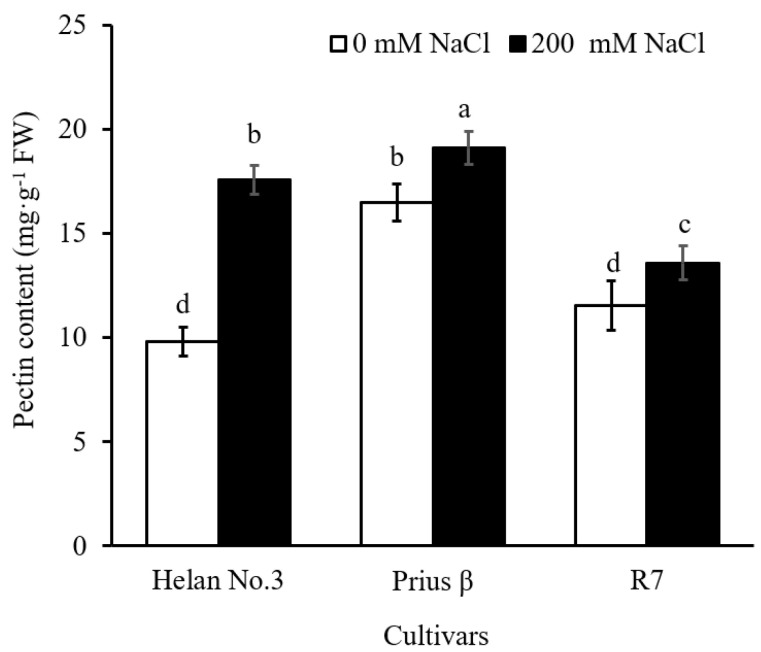
Pectin contents in root cell wall in three cultivars, Helan 3, Prius β and R7, under 0 and 200 mM NaCl treatments. Data are mean ± S.E. (*n* = 6). Different letters indicate significant differences (*p* < 0.05).

Salinity treatment significantly increased the pectin content of the root cell wall in all cultivars (Figure 3). The molar proportion of each monosaccharide component in the pectin across the cultivars is shown in Table 1. Salinity increased the molar proportion of rhamnose, arabinose and galactose in the pectin of Helan 3 and R7, while the molar proportion of uronic acid in the cultivars decreased (Table 1). In the Prius β cultivar, the molar proportion of the monosaccharide compositions had no significant changes in all monosaccharide components under salinity stress (Table 1).

Table 2 shows the effect of salt treatment on pectin characteristics, including the degree of pectin methyl-esterification (PMD) in pectin fractions, he HG:RG-I ratio, galactan side-chain length and arabinangalactan side-chain length. In the R7 cultivar, the PMD was decreased significantly and the length of galactan and arabinangalactan side chains were significantly increased when exposed to salinity, while there were no significant changes in Helan 3 and Prius β cultivars (Table 2). The HG:RG-I ratio was significantly decreased in Helan 3 and R7 cultivars, with no significant change in the Prius β cultivar (Table 2).

Root growth was negatively correlated with pectin content, E_0_ and root diameter across the cultivars (Table 3). Both E_0_ and η_N_ in Helan 3 and R7 were significantly correlated positively and negatively with pectin content and molar proportion of uronic acid in the pectin, respectively. The PMD had a significant positive correlation with root length and a negatively significant relationship with E_0_ and η_N_ in R7, relative to the other two cultivars. (Table 3).

## 3. Discussion

### 3.1. Root Growth

Spinach belongs to a broad family of Amaranthaceae, which shows relatively high salt tolerance. However, due to different cultivars selected, differences in salt tolerance among cultivars were also reported [17,18,19,20]. The root growth of Helan 3 and Prius β was more affected by salinity; however, R7 showed higher root growth [21]. This indicated that the R7 cultivar had higher salt tolerance than the other two cultivars. Root growth could be affected by various factors under salinity. Pectin is one of these factors which has been reported to regulate root growth [22]. The polysaccharides, degree of esterification, HG:RG-I ratio and neutral side chains of pectin may all affect cell elongation and root growth because these pectin constituents affect cation binding, pH adjustment and ion homeostasis in the cell wall [17,21,22,23]. Under salinity stress, all cultivars showed a siginificant increase in root diameter. This may be due to a pH decrease in apoplasts [24,25]. In barley, a decrease in pH resulted in an increase in rhizodermal cell diameter [24], thereby thickening the roots. The correlation analysis was consistent with these results (Table 3).

### 3.2. Root Cell Wall Extensibility and Viscosity

Cell wall extensibility is known to regulate cell elongation. This extensibility is associated with cell wall structure and composition [26,27]. It was recently reported that salinity stress could alter the cell wall structure and composition, thereby affecting wall extensibility [28]. A higher cell wall extensibility is favorable for root growth under saline conditions [29]. In this study, the low E_0_ (i.e., high extensibility) in R7 compared with Helan 3 may have contributed to its higher root growth under salinity. The negative correlation between E_0_ and root length across the cultivars was indicative that cell wall extensibility in the root elongation zone is important for root growth under saline conditions (Figure 2, Table 3).

Under salinity stress, cell volume shrinkage and cell wall deformity occurred through increased cell wall synthesis and strength [17,30,31,32]. Reboul et al. [33] reported that the lack of glucuronic acid could limit cell wall expansion. Similarly, Zdunek et al. [34] reported that the amount of uronic acid in cell walls may be related to its stiffness, especially in pear plants. In this study, pectin content, the molar proportion of uronic acid in pectin and HG:RG-I ratio were significantly correlated to E_0_ under salinity stress in Helan 3 and R7 cultivars. These correlations indicate that cell wall extensibility was affected by pectin in Helan 3 and R7 cultivars, which may have affected cell expansion under the stress condition. Moelants et al. [35] reported that pectin viscosity was affected by the polysaccharide chain structure in carrot and tomato. This is also in line with the report of Mierczyńska et al. [36] that pectin viscosity is related to uronic acid content, and a smaller pectin molecule could lead to increased viscosity in carrot. Furthermore, Pieczywek et al. [37] reported an increase in GalA (galacturonic acid) content that led to softening of the cell walls. The molar proportion of uronic acid in pectin and the HG:RG-I ratio were significantly correlated with η_N_ across the cultivars; this is an indication that uronic acid in pectin and the RG-I backbone may have regulated cell wall viscosity during salinity stress in spinach.

### 3.3. Chemical Composition of Pectin

The pectin content was significantly increased across the cultivars under salinity stress (Figure 3), whereas the molar proportion of uronic acid in pectin was significantly decreased in Helan 3 and R7 cultivars (Table 1). There was a positive correlation between the molar proportion of uronic acid in pectin and the root length of the two cultivars (Table 3). This showed that pectic uronic acid was consistent with root growth in the two cultivars. A previous study reported that uronic acid in pectin had been found to provide cation binding sites, which can trap Na^+^ to reduce cellular damage [5,14]. Interestingly, combined Na^+^ and uronic acid was found to release H^+^, which adjusted the pH of apoplasts and consequently altered the expansion, thereby affecting cell wall extensibility and thus improving root growth [23,25]. In this study, no significant effect of uronic acid on root growth was found in all three cultivars. A similar trend was observed in *Suaeda salsa* and *S. oleracea* ‘Akinokagayaku’ [17], which belonged to the same family of Amaranthaceae. The role of uronic acid under salinity stress may be said to be species-dependent.

In the R7 cultivar, the PMD decreased significantly but did not show any significant difference in the other two cultivars. The PMD was significantly correlated to root length, pectin content, E_0_ and η_N_ in R7, relative to the other cultivars. This is indicative that salt tolerance in plant cultivars could be regulated by the demethylation of pectin. Zheng et al. [38] and John et al. [39] disclosed that Na^+^-induced de-esterification of pectin could result in the formation of an egg-box structure with divalent cations in the form of a gel, and the concentration of sodium ions affects the crosslinking strength. The increased galactan and arabinangalactan side chains under salinity stress in R7 may improve network formation. Pectin gels can be embedded in cellulose–hemicellulose networks and contribute to cell wall elasticity [15], which may possibly be achieved through the amount of pectin gel on cell wall hydration and the demethylation of pectin [15,40,41,42]. The degree of pectin methy-lesterification (PMD) determines negative charges and has a close negative correlation with ion adsorption in the cell walls of plant roots [43,44]. The increases in cell wall elasticity also correlated with the PMD [22]. The positive correlation between the PMD and root length in R7 indicated that the extensive demethylation of pectin enhanced salt tolerance, and the correlation between the PMD and E_0_ in the R7 cultivar could be attributed to the decrease in the PMD that correlated negatively with the cell wall extensibility. The increased side chains and demethylation of pectin under salinity stress may be the reason for the higher salt tolerance of R7 compared to Prius β and Helan 3.

The molar proportion of uronic acid of pectin and the HG:RG-I ratio were found to be significantly correlated to cell viscosity. The RG-I backbone is reportedly involved in the regulation of the water-binding capacity of potato cell walls [45]. The viscosity of cell walls in apple plants was also reported to affect its water-binding capacity [46]. This cell wall viscosity could be regulated by the water-binding capacity provided by pectin characteristics (HG:RG-I ratio). In Broxterman and Schols [47], pectin and cellulose were reported to have been linked by short and highly branched galactose and arabinose side chains on the RG-I backbone. Therefore, salinity stress increased the length of the galactose and arabinose side chains in R7 (Table 2), while they were decreased substantially in Helan 3 and Prius β cultivars (Table 2). The increased length of the galactose and arabinose side chains under salinity stress may provide more binding sites for pectin and cellulose, thereby increasing cell wall stability. This may possibly benefit the salt tolerance in R7.

## 4. Materials and Methods

### 4.1. Plant Materials

The seeds of *Spinacia oleracea* L., salt-sensitive ‘Helan 3′ (bred in Holland) and ‘Prius β’ (bred in Denmark), and salt-tolerant ‘R7’ (bred in Japan) were purchased from a seed market in the city of Tottori, Japan. The evaluation of their salt tolerance was based on the effects of salinity stress on root length, which is an important indicator for evaluating salt tolerance [48,49]. Spinach seeds were washed and soaked in distilled water for 24 h. Seed germination and seedling growth were conducted in growth chambers (MLR-350HT; Sanyo, Osaka, Japan) at 20 °C. Fifteen seeds were aligned on a sheet of filter paper in a zip-lock plastic bag. Filter papers were moistened every day during the three-day germination period for spinach in the dark. After germination, 1/12 diluted Hoagland solution, containing 0 and 200 mM NaCl treatments, was applied to the roots every 2 days (d). Seedlings were subjected to salinity treatments for 6 d. Light conditions were set to 12/12 h cycles (day/night). Each salt concentration treatment comprised 24 filter paper germination sheets. At the end of germination test, six filter papers were randomly selected from each treatment combination for the measurement of the root length of each seedling.

### 4.2. Mechanical Parameters of the Root Cell Wall

Root samples separated from cultured seedlings were excised 10 mm from the apical zone and immediately transferred to boiling methanol in a water bath (80 °C, 5 min). Methanol-killed root segments were rehydrated with 1/12 diluted Hoagland solution (pH 6.5) and extended. We determined the root extensibility following Tanimoto et al. [29]. The cell wall extensibility and viscosity were measured using a creep meter (RE2-33005C-1,2; Yamaden, Tokyo, Japan). A 3–7 mm root segment behind the root cap was fixed between the two clamps of the creep meter used for the measurement of extension. Roots were stretched under 0.1 N tensile force for 5 min and then released for 5 min. The final length at 5 min was read as the reversible extension (elastic extension), while the length difference between final length and original length (4 mm) was read as the plastic extension. The elastic modulus (E_0_) and the viscosity coefficient (η_N_) were determined using the software supplied with the creep meter, which indicated the extensibility and viscosity, respectively [29]. An increase in E_0_ value indicated a decrease in elasticity, while greater η_N_ values indicated higher cell wall viscosity [29]. We measured the cell wall physical parameters of at least 18–25 root segments for each replicate.

### 4.3. Extraction of Cell Wall Fractions

Seedling roots were taken out from growth bags, thoroughly washed with distilled water and cut into 10 mm segments behind the root tips as elongation zones [50]. About 50 segments from 4 filter paper sheets were taken as one replicate, while 6 replicates were measured for one treatment.

Cell wall pectin was extracted using the procedure in An et al. [6]. The root segments were immediately homogenized in a mixture of ice-cold Tris-HCl buffer (pH 7.4) and Tris buffer-saturated phenol using a bead crusher (Model μT-12; TAITEC Co., Ltd., Tokyo, Japan). The homogenate was centrifuged at 3800× *g* for 10 min at 10 °C. The supernatant was discarded and the pellet containing the cell walls was further purified by sequential incubation and centrifugation in ethanol, acetone, a mixture of methanol: chloroform (1:1, *v*/*v*), and again in acetone and ethanol. The centrifuged residues were designated as cell walls after treated with pronase in phosphate buffer (pH 7.0). The pectin fractions were extracted five times with CDTA at pH 6.5 at 20 °C. To extract the remaining polyuronides, cell walls were further extracted three times with CDTA at 100 °C (hot CDTA) for 1 h each. These CDTA extractions were designated as the pectin fraction.

### 4.4. Characterization of the Extracts

#### 4.4.1. Sugar Composition

The amounts of total sugars and uronic acid in each extract of cell wall were measured using the phenol–sulfuric acid method [51] and *m*-hydroxydiphenyl colorimetric method [52], respectively.

The pectin fraction was hydrolyzed with 4 M trifluoroacetic acid at 100 °C for 6 h in a sealed tube. Excess trifluoroacetic acid was removed by evaporation under reduced pressure. Neutral monosaccharides (Rhamnose, Arabinose, Xylose, Mannose, Glucose and Galactose) in pectin were derivatized and analyzed as their acetylated derivatives using gas chromatography (GC) [53]. The pectin fraction was hydrolyzed with 4 M trifluoroacetic acid at 100 °C for 6 h in a sealed tube. Trifluoroacetic acid was removed by evaporation under reduced pressure (Speedvac SPD131DDA, Thermo Scientific, Waltham, MA, USA). 5 mg ammonium hydrochloride and 0.5 mL pyridine were added and allowed to react in a 90 °C water bath for 30 min. Acetic anhydride (0.5 mL) was added to the test tube and incubated at 90 °C for another 30 min to allow the acetylation reaction to occur. The acetylated derivatives were analyzed by GC (GCMS-QP2010C Plus, SHIMADZU, Kyoto, Japan) with a HP-5MS column (0.25 mm × 30 m × 0.25 μm) and a flame ionization detector. The temperature program was set at 130 °C and maintained for 5 min and then increased to 240 °C at an increment of 5 °C/min. The 240 °C temperature was held for 5 min. The HG:RG-I ratio was calculated by GalA/Rha; the side-chain length of galactan and arabinogalactan were Gal/Rha and (Ara + Gal)/Rha in mol%, respectively [11].

#### 4.4.2. Determination of Pectin Methyl-Esterification

The degree of pectin methyl-esterification (PMD) was quantified by the amount of methanol produced using enzymatic pectin hydrolysis and the colorimetric method as described in Anthon et al. [54]. Pectin samples were mixed with alcohol oxidase 30 °C in a water bath. After 10 min, freshly prepared 5 mg/mL Purpald in 0.5M NaOH was added, and the mixture incubated for an additional 40 min at 30 °C. Methanol content was determined at 550 nm absorbance using a UV–visible spectrophotometer (Shimadzu UV−1900i, Shimadzu, Tokyo, Japan). The PMD was calculated as the moles of methyl ester groups per 100 mol of uronic acid.

### 4.5. Statistical Analysis

All data were analyzed using the analysis of variance (ANOVA) and correlation; means were compared using Tukey’s Honestly Significant Difference test (*p* < 0.05). All statistical analyses were performed using SPSS software version 28.0 (SPSS, Inc., Chicago, IL, USA).

## 5. Conclusions

The cell wall pectin played important roles in regulating root growth and root diameter under salinity stress. Pectin can affect cell wall viscosity, which may be related to the molar proportion of uronic acid or the HG:RG-I ratio in spinach cultivars. In comparing Helan 3 and Prius β cultivars, the high salt tolerance of the R7 cultivar was significantly correlated with the pectin characteristics. The demethylation and increased side chains of pectin under salinity stress may lead to changes in cell wall elongation, and thus root growth, which fundamentally enhances plant growth under salt tolerance.

## Figures and Tables

**Table 1:** Monosaccharide composition (mol%) of rhamnose (Rha), arabinose (Ara), xylose (Xyl), mannose (Man), glucose (Glc), galactose (Gal) and uronic acid (UA) in Helan 3, Prius β and R7 cultivars under salinity stress. Data are mean ± S.E. (*n* = 6).

Cultivar	NaCl (mM)	Mol%						
		Rha	Ara	Xyl	Man	Glc	Gal	UA
Helan 3	0	5.9 (0.4) c	9.2 (0.6) b	4.3 (0.4) a	2.4 (0.2) b	8.1 (1.3) a	14.7 (0.8) c	55.5 (2.1) a
	200	7.5 (0.3) b	12.1 (0.7) a	5.1 (0.5) a	4.0 (0.6) b	5.1 (1.1) ab	17.9(1.2) ab	48.3 (2.3) b
Prius β	0	6.2 (0.7) c	11.1(1.3) ab	3.1 (0.5) b	9.7 (1.2) a	2.7 (0.5) b	19.8 (2.1) a	47.4 (3.9) b
	200	6.2 (0.4) c	9.8 (0.6) ab	2.1 (0.2) bc	11.2 (0.9) a	1.7(0.2) b	17.6(1.2) ab	51.4(3.1) ab
R7	0	8.8 (0.8) b	10.6 (0.9) b	2.4 (0.3) bc	1.3 (0.1) b	4.2 (0.5) b	14.6 (1.0) c	58.2 (2.6) a
	200	10.4 (0.7) a	12.5 (0.8) a	1.5 (0.3) c	2.5 (0.2) b	5.1 (0.7) ab	20.9 (1.3) a	47.0 (2.9) b

Means followed by the same letter in the same column are not significantly different (*p*  <  0.05).

**Table 2:** Degree of pectin methyl-esterification (PMD) in pectin fractions, HG:RG-I ratio, galactan side-chain length and arabinangalactan side-chain length in Helan 3, Prius β and R7 cultivars under salinity stress. Data are mean ± S.E. (*n* = 6).

Cultivar	NaCl (mM)	PMD (%)	HG:RG-I Ratio	Galactan Side Chain	Arabinangalactan Side Chain
Helan 3	0	35.1 (6.7) c	9.8 (1.1) a	2.6 (0.3) ab	4.2 (0.3) b
	200	31.4 (2.8) c	6.5(0.7) bc	2.4 (0.1) bc	4.0 (0.1) bc
Prius β	0	42.4 (3.5) b	8.3 (1.5) ab	3.3 (0.3) a	5.0 (0.3) a
	200	38.8 (3.2) bc	8.6 (1.1) ab	2.8 (0.1) ab	4.4 (0.1) ab
R7	0	59.0 (6.9) a	7.0 (0.8) ab	1.7 (0.1) d	2.9 (0.1) d
	200	42.5 (2.2) b	4.7 (0.6) c	2.0 (0.1) c	3.2 (0.1) c

Means followed by the same letter in the same column are not significantly different (*p*  <  0.05).

**Table 3:** Cross-correlation coefficients of final root length, elastic moduli (E_0_), viscosity coefficient (η_N_), pectin content in cell wall, molar proportion of uronic acid in pectin, HG:RG-I ratio and degree of pectin methyl-esterification (PMD) of root cell wall in spinach under salinity stress.

	Root Length	E_0_	η_N_	Pectin Content	Uronic Acid	HG:RG-I Ratio	PMD
**Helan 3**							
E_0_	−0.875 **						
η_N_	−0.868 **	0.780 **					
Pectin content	−0.885 **	0.685 *	0.825 **				
Uronic acid	0.601 *	−0.656 *	−0.623 *	−0.268			
HG:RG-I ratio	0.621 *	−0.556	−0.638 *	−0.640 *	0.325		
PMD	0.247	0.006	−0.355	−0.395	0.063	0.352	
Root diameter	−0.889 **	0.591 *	0.740 **	0.927 **	−0.344	−0.579 *	−0.412
**Prius β**							
E_0_	−0.628 *						
η_N_	0.275	−0.264					
Pectin content	−0.712 **	0.384	0.180				
Uronic acid	−0.175	0.114	−0.869 **	−0.291			
HG:RG-I ratio	0.013	0.098	−0.838 **	−0.408	0.964 **		
PMD	0.227	0.057	−0.185	−0.235	0.056	0.056	
Root diameter	−0.659 *	0.286	−0.611 *	0.586 *	0.363	0.363	−0.086
**R7**							
E_0_	−0.816 **						
η_N_	−0.821 **	0.879 **					
Pectin content	−0.737 **	0.689 *	0.741 **				
Uronic acid	0.708 **	−0.611 *	−0.666 *	−0.557			
HG:RG-I ratio	0.615 *	−0.633 *	−0.657 *	−0.512	0.979 **		
PMD	0.807 **	−0.657 *	−0.651 *	−0.581 *	0.450	0.351	
Root diameter	−0.914 **	0.779 **	0.786 **	0.823 **	−0.656 *	−0.585 *	−0.687 *

* Correlation is significant at the 0.05 level (two-tailed); ** Correlation is significant at the 0.01 level (two-tailed). (*N* = 12).

## Data Availability

Data sharing is not applicable to this article.
